# The Wearable Cardioverter-Defibrillator: Experience in 153 Patients and a Long-Term Follow-Up

**DOI:** 10.3390/jcm9030893

**Published:** 2020-03-24

**Authors:** Stephanie L. Rosenkaimer, Ibrahim El-Battrawy, Tobias C. Dreher, Stefan Gerhards, Susanne Röger, Jürgen Kuschyk, Martin Borggrefe, Ibrahim Akin

**Affiliations:** 1 First Department of Medicine, Medical Faculty Mannheim, University Heidelberg, 68167 Mannheim, Germany; Stephanie-Luise.Rosenkaimer@umm.de (S.L.R.); tobias.dreher@yahoo.com (T.C.D.); Stefan.gerhards@umm.de (S.G.); Susanne.roeger@umm.de (S.R.); Juergen.Kuschyk@umm.de (J.K.); martin.borggrefe@umm.de (M.B.); ibrahim.akin@umm.de (I.A.); 2 DZHK (German Center for Cardiovascular Research), Partner Site, Heidelberg-Mannheim, 68167 Mannheim, Germany

**Keywords:** WCD, life-vest, sudden cardiac death

## Abstract

Background: The wearable cardioverter-defibrillator (WCD) is available for patients at high risk for sudden cardiac death (SCD) when immediate implantable cardioverter-defibrillator (ICD) implantation is not possible or indicated. Patient selection remains challenging especially in primary prevention. Long-term data on these patients is still lacking. Methods: 153 patients were included in this study. They were prescribed the WCD between April 2012 and March 2019 at the University Medical Center, Mannheim, Germany. The mean follow-up period was 36.2 ± 15.6 months. Outcome data, including all-cause mortality, were analyzed by disease etiology and ICD implantation following WCD use. Results: We analyzed 56 patients with ischemic cardiomyopathy, 70 patients with non-ischemic cardiomyopathy, 16 patients with prior need for ICD/CRT-D (device for cardiac resynchronization therapy with defibrillator) explanation, 8 patients with acute myocarditis and 3 patients with congenital diseases. 58% of the patients did not need ICD/CRT-D implantation after WCD use. 4% of all patients suffered from appropriate WCD shocks. 2 of these patients (33%) experienced appropriate ICD shocks after implantation due to ventricular tachyarrhythmias. Long-term follow-up shows a good overall survival. All-cause mortality was 10%. There was no significant difference between patients with or without subsequent ICD implantation (*p* = 0.48). Patients with ischemic cardiomyopathy numerically showed a higher long-term mortality than patients with non-ischemic cardiomyopathy (14% vs. 6%, *p* = 0.13) and received significantly more ICD shocks after implantation (10% of ischemic cardiomyopathy (ICM) patients versus 3% of non-ischemic cardiomyopathy (NICM) patients, *p* = 0.04). All patients with ventricular tachyarrhythmias during WCD use or after ICD implantation survived the follow-up period. Conclusion: Following WCD use, ICD implantation could be avoided in 58% of patients. Long-term follow-up shows good overall survival. The majority of all patients did not suffer from WCD shocks nor did receive ICD shocks after subsequent implantation. Patient selection regarding predictive conditions on long-term risk of ventricular tachyarrhythmias needs further risk stratification.

## 1. Introduction

The wearable cardioverter defibrillator (WCD), manufactured by ZOLL (Pittsburgh, PA, USA) is available for patients at high risk of sudden cardiac death (SCD). There is general acceptance for its use in circumstances when there is an implantable cardioverter-defibrillator (ICD) indication and concomitant but temporary contraindication for immediate implantation according to the 2015 European Society of Cardiology guidelines for the management of patients with ventricular arrhythmias and the prevention of sudden cardiac death [1].

Risk stratification for SCD is mainly based on impaired left ventricular ejection fraction (LVEF) ≤ 35% [2]. Left ventricular dysfunction and heart failure are among the most powerful predictors of SCD post myocardial infarction (MI) [3,4]. In patients with newly diagnosed heart failure or in patients soon after MI with or without myocardial revascularization therapy, LVEF may improve after time especially with optimal medical treatment (OMT). This has an impact on decision making regarding persistent risk of sudden cardiac death and need for ICD implantation over time. Patients with impaired LVEF ≤ 35% were at highest risk for SCD especially in the first 30 days after MI. After a few months this risk drops progressively [5]. Despite the risk, studies such as The Immediate Risk Stratification Improves Survival Trial (IRIS) and the Defibrillator Implantation Early after Myocardial Infarction Trial (DINAMIT) have shown, that early ICD implantation after MI does not improve overall survival [6,7]. Current heart failure guidelines recommend the establishment of OMT for at least 3 months before reevaluation for persistent need of ICD implantation [2]. Recent data even suggest a prolonged period of more than 3 months with OMT before ICD implantation in patients with newly diagnosed non-ischemic cardiomyopathy (NICM) and ICM due to the possibility of delayed improvement of LVEF [8,9]. In the context of acute peripartum cardiomyopathy (PPCM), as a pregnancy-associated acute myocardial dysfunction, it is assumed that about a quarter of deaths are caused by ventricular tachyarrhythmias, mostly occurring during the first 6 months [10]. Several smaller publications reported recovery of LVEF in at least 50% of patients within 6 months after diagnosis. The majority of women demonstrated recovery without major events or persistent severe cardiomyopathy 12 months post-partum [11,12]. A retrospective study showed complete recovery of LVEF in 23% of patients and partial recovery in another 19% over a prolonged period of time, confirming frequent delayed recovery over a 6 months period in this special patient population (up to 83%) [13]. A practical guidance for prevention of SCD in these women is published in 2016 by the Heart Failure Association of the European Society of Cardiology [14].

Due to the heterogeneity of underlying heart diseases and the potential for LVEF recovery, prolonged optimal therapeutic heart failure management and treatment can be crucial. Decisions about implantation of an ICD should always be taken with caution and remain challenging in this context. Device related complications should not be left out of consideration. 

The DANISH trial investigated exclusively the benefit of an ICD implantation in patients with NICM. It was shown that these patients are at risk of ventricular tachyarrhythmias even in the chronic phase of the disease. But in this randomized trial, primary preventive ICD implantation did not reduce overall mortality of these patients [15]. But especially younger patients showed a significant reduction in all-cause mortality after ICD implantation. As a conclusion, the decision whether to implant an ICD in NICM patients should still be considered on an individual basis, considering the severity of underlying disease, progression of heart failure and concomitant arrhythmia risk versus age, life expectancy and risks of competing illness.

WCD use has been proven safe and effective [16,17,18,19]. Data published in 2015 from the WEARIT-II Registry showed that devices could be safely used. In this registry devices delivered appropriate shocks in 54% of 41 patients who experienced sustained ventricular tachyarrhythmias (sVT) during the trial. Only 0.5% (10 patients) received inappropriate shocks. Therefore it was stated in the ESC Guidelines 2015 for the management of patients with ventricular arrhythmias and prevention of SCD, that the WCD may be considered for patients with poor LVEF who are at risk of SCD for a limited period, but are not immediate candidates for an ICD (e.g., bridge to transplant, bridge to transvenous implant, peripartum cardiomyopathy, active myocarditis, and arrhythmias in early phase post MI/class IIb ) [1]. 

A one-year follow-up from WEARIT-II Registry showed a good overall survival of patients prescribed with the WCD [20]. During 12 months of follow-up 4% of the patients died. All-cause mortality showed differences between patients with ICM and NICM (4% vs. 3%). Short-term use of WCD allowed appropriate risk stratification for the decision on ICD implantation in at risk patients. Patients suffering from ventricular tachyarrhythmia events during WCD use showed a significantly higher 1-year mortality than patients without arrhythmic events (10% vs. 3%, *p*-value = 0.042).

The first randomized controlled Vest Prevention of Early Sudden Death Trial (VEST), published in 2018, failed to prove advantage for the use of WCD in patients up to 90 days after myocardial infarction and moderate to severe reduced LV-dysfunction LVEF ≤ 35% [21]. Compared to controls (*n* = 1524 with WCD and OMT, control group *n* = 778) the use of WCD did not reduce the primary outcome significantly (SCD and ventricular tachyarrhythmia death, WCD group 1.6% and control group 2.4%, *p* = 0.18). Despite the negative primary endpoint, there was a statistically significant reduction in all-cause mortality associated with the use of WCD during this period (3.1% in the WCD group vs. 4.9% in the control group, *p* = 0.04). These data remain controversial and due to several study design limitations (such as high cross-over rate and problems regarding reduced WCD wear time and patient compliance in the WCD group) it seems even more controversial and difficult to consider WCD use and selecting among all eligible patients.

Because current data remain debatable and there is a lack of data on long-term outcome of patients after termination of WCD therapy, we investigated 153 consecutive patients at our University Hospital, followed over a mean time of 36 months. We assessed long-term all-cause mortality and analyzed 3-year survival of patients with or without ventricular tachyarrhythmias during WCD use and subsequent device implantation using both electronic medical records and WCD/ICD interrogation data.

## 2. Methods

### 2.1. Patient Recruitment

This cohort study included all patients receiving a WCD at the University Medical Center, Mannheim, Germany between April 2012 and March 2019. All patients were fitted with a ZOLL LifeVest™ system (Pittsburgh, PA, USA). All patients received OMT according to the current heart failure guidelines. Each patient provided consent for the analysis of standard clinical data. The study was approved by the local ethics committee (2019-840-R) and conforms to the 1975 Declaration of Helsinki.

### 2.2. The Wearable Cardioverter-Defibrillator (WCD)

The WCD has been described previously [16,22]. The WCD programming was individually adapted to the patients underlying heart disease and electrocardiographic patterns. In general, for older patients the ventricular tachycardia (VT) zone was programmed at heart rate of 150 beats per minute (bpm) with a VT response time of 60 seconds (s) and the ventricular fibrillation (VF) zone at a rate of 200 bpm with a VF response time of 25 s. For younger and more active patients the VT zone was programmed at a heart rate of 180 bpm. First shock energy was set to the maximum output (150 J) in all patients. Any arrhythmia episode was considered as a separate episode when occurring with a minimum delay of 3 min from the previous one. Each individual episode was reviewed and classified into the following categories: Sustained VT or VF (lasting 30 s or longer) with WCD shock therapy, non-sustained VT (lasting less than 30 s), bradycardia (30 bpm or less) or asystole. Inappropriate WCD therapy was classified as non-VT/-VF episode treated by a WCD shock.

### 2.3. Baseline and Follow-Up Data Collection 

Patients were followed from the time of WCD implementation. Baseline data included the indication for WCD by disease etiology, morbidities, baseline medication, electrocardiogram data (ECG) and echocardiographic results. LVEF was calculated by using the biplane Simpson’s method, using echocardiography and/or cardiac magnetic resonance imaging (MRI). WCD was prescribed in eligible patients for at least 3 months. The prescription was based on individual risk estimation according to current guidelines. Data on arrhythmias during follow-up were prospectively collected and retrieved from ZOLL LifeVest Network™.

Long-term follow-up was counted from the day on which the patient returned the WCD. During long-term follow-up, all study-patients were clinically and echocardiographically assessed every 6 months.

Heart failure medications consisted of angiotensin converting-enzyme inhibitor/angiotensin receptor blockers (ACE-I/ARB), beta-blockers and mineralocorticoid receptor blockers (MRA) according to current heart failure guidelines [2]. Ivabradine was prescribed in selected cases in accordance with the Ivabradine and outcomes in chronic heart failure trial (SHIFT-Trial, [23]). Since 2016 selected patients received an angiotensin receptor—neprilysin inhibitor (ARNI) instead of an ACE-I or ARB. All patients were screened for iron deficiency and treated if required (intravenous iron substitution).

In cases of a missing follow-up visit, the patient, family members and/or treating physician were contacted.

### 2.4. Endpoints and Definitions

We assessed mortality rate and analyzed all-cause mortality of patients with subsequent ICD implantation and by disease etiology.

### 2.5. Statistics 

All statistical analyses were performed using IBM SPSS for Macintosh (Version 25.0. Armonk, NY: IBM Corp.). Data are presented as mean ± standard deviation or median (interquartile range) for continuous variables or as a number of cases for categorical variables expressed as frequencies and percentages. A paired *t*-test was performed for comparison of continuous variables, and a chi-squared test or, when appropriate, a Fisher’s exact test was used for comparison of categorical variables.

A *p*-value < 0.05 was considered statistically significant.

## 3. Results

### 3.1. Patients Baseline Characteristics 

**Figure 1:**
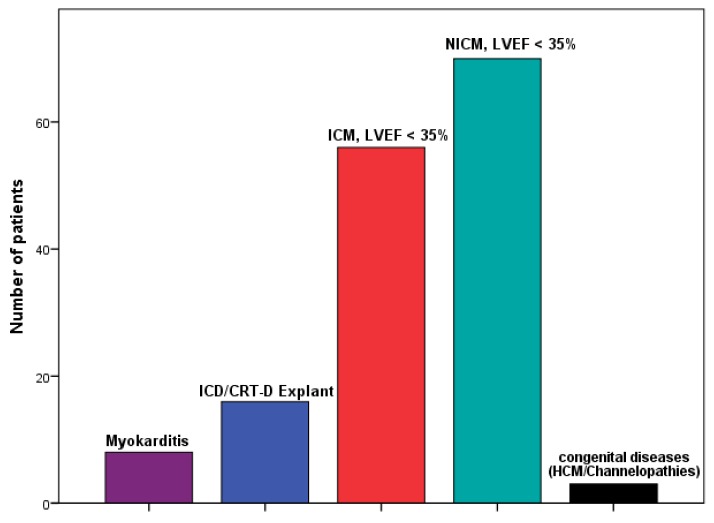
Distribution of the indication for wearable cardioverter-defibrillator (WCD) in a cohort of 153 patients according to opinion of physicians. ICD: implantable cardioverter defibrillator; CRT: cardiac resynchronization therapy; ICM: ischemic cardiomyopathy; NICM: non-ischemic cardiomyopathy; LVEF: Left ventricular ejection fraction; HCM: hypertrophic cardiomyopathy.

**Figure 2:**
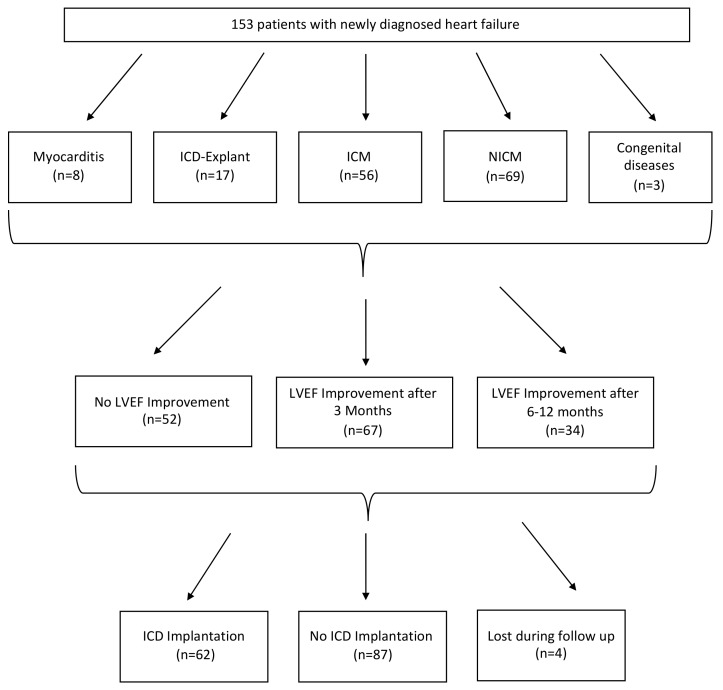
Study flow chart. ICD: implantable cardioverter defibrillator; ICM: ischemic cardiomyopathy; NICM: non-ischemic cardiomyopathy; LVEF: Left ventricular ejection fraction.

We included 153 patients receiving a WCD at our Center between April 2012 and March 2019 with a mean follow up time of 36.2 ± 15.6 months (median 34: interquartile range (IQR) 24–46 months). The most common indication for WCD use (*n* = 70, 46%) was primary preventive therapy in patients with NICM and LVEF ≤ 35% (Figure 1, Table 1). The second common indication (*n* = 56, 37%) was primary prevention therapy in patients with ICM and LVEF ≤ 35%. 11% of the patients (*n* = 16) needed the WCD because of CRT-D/ICD explanation. Further indications are shown in Figure 1. The mean age of the patients was 60 ± 14 years (Table 1) with a male predominance (female *n* = 35%). 4 patients were lost to follow-up after return of the WCD (Figure 2).

Twenty-six patients (17%) had a history of ventricular tachycardia (VT) and/or ventricular fibrillation (VF) prior to WCD use based on 12-lead ECG, holter ECG and/or Telemtry. 13 patients (8%) suffered from cardiogenic shock prior to WCD implementation. 61 patients (40%) had a history of coronary artery disease (CAD), 48 patients (31%) had a previous history of MI and 11 patients (7%) underwent myocardial bypass surgery. Episodes of atrial fibrillation/flutter were recorded in 37 patients (24%). Nine patients (6%) had a history of transient ischemic attacks (TIA) and/or stroke.

Regarding cardiovascular risk factors, arterial hypertension was documented in 87 patients (57%) and hyperlipidemia in 67 patients (44%). In addition, 106 individuals (69%) were overweight (BMI > 25 kg/m^2^) and 67 patients (44%) were active smokers. Moderately to severe chronic kidney disease (GFR < 45 mL/min)/or Dialysis was present in 20 individuals (13%).

The mean hospitalization time among all patients at baseline was 15.8 ± 11.9 days.

### 3.2. WCD Data

The average WCD wear time was 65.1 ± 42 days with a mean daily use of 21.5 ± 3.5 hours (Table 2). The main reasons for stopping WCD use were improved LVEF (*n* = 77, 50%), ICD/CRT-D implantation (*n* = 56, 37%) and incompliance/unwillingness to continue WCD use (*n* = 13%). Two patients (1%) died while wearing the WCD. The cause of death was septic shock in one patient and pulseless electrical activity and low output syndrome after hemodialysis in the other patient. Clinical observation and the ZOLL LifeVest Network™ showed no evidence of ventricular tachyarrhythmias in these two patients.

Six patients (4%) received appropriate WCD shocks. The majority had a LVEF below 35% at baseline. One female patient with active myocarditis had a preserved LVEF of 53% with documented recurrent nsVT (non-sustained ventricular tachycardia). Five patients with WCD shocks (83%) had an ischemic cardiomyopathy (ICM) with reduced LVEF below 35%. One patient with WCD shock (17%) had non-ischemic cardiomyopathy (NICM). Five patients (3%) presented with ventricular tachycardia (VT) while one patient presented with ventricular fibrillation (VF). All episodes were successfully terminated by the first WCD shock. All patients with appropriate WCD shocks underwent subsequent ICD implantation, and no patient died during long-term follow-up. Two of these 6 patients with prior WCD shock received ICD shocks due to recurrent VT afterwards. Characteristics of these patients are presented in Table S1 (Supplemental Material). One patient received an inappropriate WCD shock due to artifactual voltage fluctuations misinterpreted as ventricular arrhythmia by the WCD. The patient failed to react and push the response button of the WCD despite tactile and audible alarms of the WCD. Overall a mean of 12 shocks were actively inhibited by the patients.

### 3.3. Long-Term Follow-Up 

#### 3.3.1. Echocardiography Data 

At the end of WCD use period, mean LVEF had improved significantly from 28.61 ± 10.15% at baseline up to 36.94 ± 11.3% at 3 months (*p* < 0.001) (Table 3). After 6 to 12 months of follow-up further significant improvement of LVEF to 40.96 ± 12.68% was noted (*p* < 0.001). Among all 153 patients 67 (44%) showed improvement of LVEF after 3 months and additional 34 patients (22%) improved after 6–12 months (Table 2). In total 101 patients (66%) showed an improvement of LVEF over time. The majority of these 101 patients with improved LVEF (*n* = 56, 55%) had NICM. Forty-nine patients (32%) had no change in LVEF and another 3 patients (2%) showed a decline in LVEF. All patients were followed at least 9 months regarding changes in LVEF. 

#### 3.3.2. ECG Data

Left bundle branch block (LBBB) at baseline was present in 25 patients (16%), right bundle branch block (RBBB) in 17 patients (11%, Table 2). QRS duration at baseline was 109.81 ± 30.77 ms and did not change significantly during follow-up period (*p*-value = 0.702 for comparison of baseline versus after 3 months and *p*-value = 0.211 for comparison baseline versus after 6–12 months), also the PQ interval did not change (Table 3). QTc duration however decreased significantly from 460.04 ± 54.47 ms at baseline to 445.39 ± 40.86 ms after 3 months (*p*-value = 0.003). Therefore, the QTc time did not change after 6–12 months compared to baseline (*p*-value = 0.182).

#### 3.3.3. Rhythm

At baseline 133 patients (87%) had sinus rhythm and 12 patients had persistent atrial fibrillation (8%), 13 patients had atrioventricular block I (9%) and 4 patients had a pacemaker rhythm (3%) due to atrioventricular block III. The majority of these characteristics did not change significantly during the follow-up period (Table 3).

#### 3.3.4. Laboratory Value

Eighty-four patients presented with elevated cardiac biomarker pro-BNP (55%) at baseline. During follow-up we documented a statistically significant decline to 28% (*n* = 43 patients) after 6–12 months (*p*-value baseline versus 3 months = 0.039, *p*-value baseline versus 6–12 months = 0.001). The absolute BNP-value showed significant decrease especially during the first 3 months of heart failure therapy from 7732.63 ± 7148.99 ng/L to 2099.03 ± 3688.02 ng/L (*p* = 0.011) (Table 3).

#### 3.3.5. Device Implantation After WCD Use

The majority of patients were not implanted with an ICD/CRT-D after WCD use (*n* = 87, 58%), among these patients 2 denied implantation despite medical recommendation. Sixty-two patients (42%) had an ICD indication and were implanted. Among these patients 39 had NICM (63% of implanted patients) and 23 patients had ICM (37%). 13 patients underwent ICD re-implantation and 2 patients had myocarditis. 2 patients (1%) died before implantation. Among all implanted patients 4 received an ICD later during the follow-up period, but not directly after stopping WCD use. 

In total 13 patients (9%) received a transvenous ICD (single- or dual-chamber-ICD), 12 patients (8%) received a CRT-D and 37 patients (24%) received a subcutaneous ICD (S-ICD) (Table 2). 

#### 3.3.6. Arrhythmic Events During Long-Term Follow-Up 

8 of all implanted 62 patients (13%) received an appropriate ICD treatment after implantation (Table 2), 2 of whom had experienced appropriate WCD shocks before. All 8 patients with ICD shock had a baseline left ventricular ejection fraction (LVEF) below 35% (3 patients showed improvement of LVEF > 35% after 6–12 month). 6 of these patients had ICM (75%), and 2 patients were diagnosed with NICM (25%). Detailed characteristics of these patients are shown in Table S2 (Supplemental Material). Among all implanted patients we recorded tachyarrhythmic episodes in 16 patients (26%), in some patients’ multiple arrhythmic episodes were registered during the follow-up period. In detail we counted 10 episodes of sustained ventricular tachycardia (VT), 16 episodes of non-sustained ventricular tachycardia (nsVT) and 2 episodes of ventricular fibrillation (VF) (Table 2). None of these patients died during the follow-up period. Inappropriate ICD shocks were not observed.

The characteristics of patients with and without appropriate ICD shock are illustrated as frequency analysis in Table S4 (Supplemental Material). 

#### 3.3.7. All-Cause Mortality

During mean follow-up 15 patients died (all-cause mortality 10%). Thirteen percent of patients with implanted ICD (*n* = 8) and another 8% of patients without implanted ICD (*n* = 7) died (*p*-value = 0.48) (Table 4). 

By disease etiology 8 patients in the ICM group (14%) versus 4 patients in the NICM group (6%) died (*p*-value = 0.13) (Table 5). Patients with ventricular arrhythmia events during WCD use or after ICD implantation showed no higher long-term mortality; all of these patients survived the follow-up period. 

Further characteristics of patients who died and of those who survived the follow-up period are illustrated as frequency analysis in Table S3 (Supplemental Material). 

#### 3.3.8. Rehospitalizations during long term follow up

67% of the patients (*n* = 102) were re-hospitalized during follow-up due to cardiovascular complications (55%), congestive heart failure (7%), stroke (4%), or VT/VF (6%) (Table 3).

## 4. Discussion

Our single center study was aimed to evaluate real-world experience of ventricular tachyarrhythmia events and long-term outcome among patients with prior WCD use at high risk for SCD. In the current report we wanted to confirm and extend prior knowledge. The major findings of our study were as follows: 

WCD therapy is safe and successfully bridged all patients to either LVEF recovery or ICD implantation. WCD allowed appropriate risk stratification for decision on ICD implantation in at risk patients during short-term follow-up. Following WCD, ICD implantation could be avoided in over the half of the patients (58%). The most common reason not to implant an ICD was improvement in left ventricular function (in 66% of all patients), predominantly in patients with NICM (55% of all improved patients). In this context we showed that the majority of patients did not suffer from any kind of ventricular tachyarrhythmia events during WCD use (94%) nor did receive ICD shocks due to ventricular arrhythmias after subsequent device implantation (87%). Those patients, who suffered from appropriate shock therapy (regardless whether WCD or following ICD shocks) all survived the long-term follow-up period. Two patients of whom suffered from appropriate WCD shocks (*n* = 6) experienced also appropriate ICD shocks due to ventricular arrhythmias post implantation (33%). 

The long-term follow-up shows good overall survival of all patients, initially prescribed the WCD. All-cause mortality in our cohort was 10% over a mean follow-up period of 36.2 ± 15.6 months. There was no significant difference in all-cause mortality between patients with or without subsequent ICD implantation after WCD use (13% vs. 8%, *p* = 0.48). Patients with ICM numerically showed a higher long-term all-cause mortality than patients with NICM (14% vs. 6%, *p* = 0.13) and received significantly more appropriate ICD shocks due to ventricular arrhythmic events after implantation (10% of ICM patients vs. 3% of NICM patients, *p* = 0.04). Regarding the safety and effectiveness of the WCD we reported reliable termination of arrhythmic episodes such as VT/VF (*n* = 6) according to previous data [16,19,24]. One inappropriate shock was registered due to motion artifacts and inappropriate use of the WCD. 

The main reason for stopping WCD use was improved left ventricular function in 44% of cases after 3 months (*n* = 67 of all patients). Thirty-four more patients (22%) showed further or delayed improvement of LVEF even after 6–12 months. Especially due to delayed LVEF recovery in a reasonable number of patients we are convinced, that a prolonged wearing time of WCD should be discussed on individual basis to avoid premature or even unnecessary ICD implantation. We documented a decrease of BNP levels over time, suitable to the high number of patients with improving left ventricular function under optimal guideline-based heart failure therapy. 

Our long-term follow-up shows good overall survival. 9 % of all patients (*n* = 134) were alive after the follow-up period of 36.2 ± 15.6 months, irrespective of subsequent device implantation or appropriate ICD shock therapy. Patients with ventricular arrhythmia events during WCD use or after ICD implantation were alive at the end of follow-up period. Other studies reported a significantly higher 1-year mortality in this collective [20].

Data between 2002 and 2006 still showed a 10.1% one-year mortality [16]. In 2018 data from the prospective one-year follow up of the WEARIT-II Registry [20] already showed a lower all-cause mortality during a 12 month-follow up (4%). The mortality rates in cardiac high-risk patients tend to be lower in current times, as shown in recently published data [25] and related to the findings in our study (1-year all-cause mortality 4.7%). 

This implies, that a clinical pathway, incorporating the WCD, allows implementing guideline directed heart failure therapy, even in prolonged recovery periods. It helps raising awareness for patients at persistent high-risk for sudden cardiac death and permits individualized timing of an ICD implantation. With the help of the WCD unnecessary or premature device implantations with possible harmful side effects for the patient can be avoided. 

Among all our implanted patients (*n* = 62), the minority suffered from reported shocks or arrhythmic episodes during long-term follow-up (shocks *n* = 8, 13%). Only 2 of these 8 patients had prior appropriate WCD shocks due to arrhythmic events. The majority of successfully rescued patients with appropriate WCD shocks (5/6) or later ICD shocks (6/8) were diagnosed with ICM. 

Several publications have also reported higher WCD shock rates in patients with newly diagnosed ICM than in patients with newly diagnosed NICM [17,26,27]. Nevertheless, there are data showing a relevant arrhythmic risk in patients with newly diagnosed NICM and non-optimized heart failure therapy [9] and there are data that also confirmed the risk of life threatening ventricular arrhythmias in the chronic phase of NICM [15]. During our long-term follow-up, 2 implanted patients with NICM received appropriate ICD therapy due to ventricular tachycardia. Both patients survived the long-term follow-up period. 

The Vest Trial (21) as the first randomized controlled trial did not reveal any significant differences in the primary endpoint, namely reduction in sudden cardiac death in patients up to 90 days after myocardial infarction and moderate to severe reduced LV-dysfunction (LVEF ≤ 35%) and should be interpreted as negative (WCD group 1.6% and control group 2.4%, *p* = 0.18). On the other hand, there was a statistically significant reduction in all-cause mortality associated with the use of WCD during this period (3.1% in the WCD group vs. 4.9% in the control group, *p* = 0.04). The data interpretation remains difficult and controversial due to several study design limitations such as a high cross-over rate in both groups and a fairly low daily WCD wear time (in the WCD group hours per day WCD worn: 14.1). Among the 48 participants in the WCD group who died, only 12 had worn the WCD at time of death. In an as-treated analysis, a significantly lower percentage of patients died when wearing the WCD as recommended. 

Taken all these data and results into count, the WCD remains a feasible and safe strategy for cardiac high-risk patients, if compliance and understanding for the device is given. But it seems reasonable, that more studies and larger registries will be necessary to further optimize risk stratification in these patients. It is necessary to investigate and identify predictive conditions for patients at especially long-term risk for ventricular arrhythmias and SCD beyond left ventricular dysfunction and symptoms of heart failure. 

### Limitations of the Study

Our study has several limitations. This was a single center study including a variety of patient conditions. The number of patients in our study was too small to permit conclusions about predictive factors for arrhythmias, shocks, and death. Not all patients received detailed systematic ECG documentation during each follow-up visit for further analysis. We did not evaluate other cardiac imaging modalities such as MRI data (regarding scar or fibrosis) to better describe and identify patients at risk for arrhythmias and SCD. The prescription of WCD was based on individual patient risk. In most of the death-cases during the follow-up period, detailed postmortem data regarding cause of death or ICD reports were not available and could not be analyzed. 

## 5. Conclusions

WCD therapy is proven to be safe and allowed appropriate risk stratification for decision on ICD implantation in at risk patients during short-term follow-up. Following WCD, ICD implantation could already be avoided in 58% of our patients. The most common reason not to implant an ICD was improvement in left ventricular function. Especially due to delayed LVEF recovery in a reasonable number of patients, a prolonged wearing time of WCD should be discussed on individual basis to avoid premature or even unnecessary ICD implantations. Especially in patients with NICM decision making remains challenging and current data remain controversial. The long-term follow-up shows good overall survival of all patients, initially prescribed the WCD. There was no significant difference between patients with or without subsequent ICD implantation. This implies, that current decision making, and implanting strategies are reasonable. But on the other hand, the majority of patients did not suffer from any kind of malignant arrhythmic episodes during WCD use nor did receive ICD shocks post device implantation. 

So, we conclude, that even with the help of WCD protection and monitoring function, patient selection regarding predictive conditions on especially long-term risk of ventricular arrhythmias and future benefit of ICD implantation needs further examination, optimization and specification. Most likely with the help of additional multimodal diagnostic strategies and larger studies or registries.

## Figures and Tables

**Table 1:** Baseline characteristics of 153 patients with wearable cardioverter defibrillator (WCD).

Variables	(*n* = 153)
**Demographics**	
Age, mean ± SD *(years)	60 ± 14
Female, *n* (%)	35 (23)
**Indication for WCD use, *n* (%)**	
Myocarditis	8 (5)
ICD */CRT-D * Explant	16 (10)
ICM *, LVEF * < 35%	56 (37)
NICM *, LVEF < 35%	70 (46)
Congenital diseases	3 (2)
**Clinic parameter**	
Cadiogenic shock at event, *n* (%)	13 (8)
Pulmonary edema, *n* (%)	14 (9)
Days of hospitalization, mean ± SD	15.8 ± 11.9
Former significant VT */VF * prior to WCD use, *n* (%)	26 (17)
**Medical history, *n* (%)**	
Former CIED explanted	7 (5)
History of CAD *	61 (40)
History of myocardial infarction	48 (31)
History of CABG *	11 (7)
Moderately severe CKD/Dialysis *	20 (13)
History of CHF *	23 (15)
History of Atrial fibrillation/flutter	37 (24)
History of TIA/stroke *	9 (6)
**Cardiovascular Risk Factors, *n* (%)**	
Smoking	67 (44)
Diabetes mellitus	31 (20)
Overweight (BMI > 25 kg/m^2^) *	106 (69)
Lipidemia	67 (44)
Hypertension	87 (57)
COPD *	16 (11)
Family history of cardiovascular disease	42 (28)

* SD: Standard deviation; ICD: implantable cardioverter defibrillator; CRT: cardiac resynchronization therapy; LVEF: Left ventricular ejection fraction; ICM: ischemic cardiomyopathy; NICM: non-ischemic cardiomyopathy; VT: ventricular tachycardia; VF: ventricular fibrillation; CAD: coronary artery disease; CABG: coronary artery bypass graft; CKD: chronic kidney disease; CHF: congestive heart failure; TIA: transient ischemic attack; BMI: body-mass-index; COPD: chronic obstructive pulmonary disease; CIED: cardiovascular implantable electronic device.

**Table 2:** Follow up data of patients during and after wearable cardioverter defibrillator (WCD) use.

Variables	(*n* = 153)
**Wear time of WCD**	
Average wear time, h/day	21.45 ± 3.52
Wear days, mean ± SD *	65.1 ± 42
Wear days, Median (IQR *)	60 (30–91)
**WCD Shocks**	
Appropriate shock, *n* (%)	6 (4)
Inappropriate shock, *n* (%)	1 (0.7)
Number of inhibitions, mean ± SD	12.28 ± 33
**Arrhythmic episodes during WCD use (*n* = 153), *n* (%)**	
None	143 (94)
Ventricular tachycardia	5 (3)
Ventricular fibrillation	1 (0.7)
Others	4 (2)
**Reason for stopping WCD use, *n* (%)**	
Improved LVEF *	77 (50)
Cardiac electronic device implantation	56 (37)
Incompliance	12 (8)
Death ^1^	2 (1)
Decision pending	1 (0.7)
Other reasons	5 (3)
**Echocardiography data**	
Not changed LVEF, *n* (%)	49 (32)
Declined LVEF, *n* (%)	3 (2)
Improved LVEF in first 3 months, *n* (%)	67 (44)
Improved LVEF after 6–12 months, *n* (%)	34 (22)
**Bundle Branch Block, *n* (%)**	
None	108 (71)
LBBB *	25 (16)
RBBB *	17 (11)
Sinus arrest/complete AV-Block	2 (1)
Fascicular block	1 (0.7)
**Device implantation after WCD use, *n* (%)**	
No	87 (58)
Yes	62 (42)
Died before implantation ^1^	2 (1)
Patient denied	2 (1)
**Device type, *n* (%)**	
None	87 (58)
Transvenous ICD *	13 (9)
CRT-D *	12 (8)
S-ICD *	37 (24)
**Reported shocks post device implantation, *n* (%)**	
No	54 (87)
Yes	8 (13)
**Arrhythmic episodes post device implantation, *n* (%)**	
Sustained VT *	10 (16)
VF *	2 (3)
Non sustained VT *	16 (26)
**Death during follow up period, *n* (%)**	
No	134 (90)
Yes	15 (10)
**Rehospitalization over mean follow up, more than one reason possible, *n* (%)**	
No	41 (27)
Yes	102 (67)
Unknown	10 (7)
Cardiovascular cause	84 (55)
Congestive heart failure	10 (7)
Atrial fibrillation	2 (1)
Stroke cause	6 (4)
VT/VF * cause	9 (6)
Any other cause	38 (25)

* SD: Standard deviation; IQR: interquartile range; LVEF: left ventricular ejection fraction; LBBB: left bundle branch block; RBBB: right bundle branch block; ICD: implantable cardioverter defibrillator; S-ICD: subcutaneous implantable cardioverter defibrillator; CRT: cardiac resynchronization therapy; VT: ventricular tachycardia; VF: ventricular fibrillation;.^1^ death due to septic shock and terminal heart failure.

**Table 3:** Follow up clinical data of patients with wearable cardioverter defibrillator (WCD), *n* = 153.

Variables	Baseline	After 3 Months	After 6–12 Months	*p* Value for Comparison	
**Echocardiography Data**				Baseline vs. 3 months	Baseline vs. 6–12 months
LVEF *, mean ± SD *	28.61 ± 10.15	36.94 ± 11.3	40.96 ± 12.68	< 0.001	< 0.001
**ECG Data, mean ± SD**					
QRS duration (ms)	109.81 ± 30.77	107.70 ± 27.53, *n* = 124	109.36 ± 28.03, *n* = 122	0.702	0.211
QTc duration (ms)	460.04 ± 54.47	445.39 ± 40.86, *n* = 124	449.55 ± 39.92, *n* = 122	0.003	0.182
PQ-interval (ms)	165.13 ± 26.13	169.86 ± 30.76, *n* = 124	164.41 ± 25.88, *n* = 122	0.946	0.797
**Rhythm, *n* (%)**					
Sinus Rhythm	133 (87)	105/124 (85)	99/122 (81)	0.639	0.127
AV-Block I	13 (9)	7/124 (6)	5/122 (4)	0.198	0.071
Pacemaker Rhythm	4 (3)	8/124 (7)	15/122 (13)	0.06	<0.01
Ventricular Fibrillation	1 (0.7)	0/124 (0)	0/122 (0)		
Atrial Fibrillation	12 (8)	10/124 (8)	8/122 (7)	0.416	1.000
**Laboratory values (ng/L)**					
Elevated Pro-BNP, *n* (%)	84 (55)	65 (43)	43 (28)	0.039	0.001
BNP, mean ± SD, (median)	7732.63 ± 7148.99	2099.03 ± 3688.02	2542.28 ± 8987.36	0.011	0.283

* LVEF: left ventricular ejection fraction; SD: standard deviation; ECG: electrocardiography.

**Table 4:** 4-year mortality by ICD implantation during median follow-up (median 34: IQR 24–46 months).

Death After	ICD Implantation, *n* = 62, (%)	No ICD, *n* = 87, (%)	*p*-Value ^1^
1 year	1 (2)	4 (5)	0.40
2 years	1 (2)	5 (6)	0.40
3 years	5 (8)	5 (6)	0.74
4 years	8 (13)	5 (6)	0.15
During WCD use	0 (0)	2/91 (2)	0.51
overall	8 (13)	7 (8)	0.48

^1^*p*-value for comparison of ICD Implantation versus No ICD Implantation; ICD: implantable cardioverter defibrillator.

**Table 5:** 4-year mortality by disease etiology during median follow-up (median 34: IQR 24–46 months).

Death After	ICM, *n* = 56 (%)	NCIM, *n* = 70 (%)	Others, *n* = 23 (%)	All, *n* = 149 (%)	*p*-Value ^1^
1 Year	2 (4)	4 (6)	1 (4)	7 (5)	0.69
2 Years	3 (5)	4 (6)	1 (4)	8 (5)	1.00
3 Years	6 (11)	4 (6)	1 (4)	11 (7)	0.34
4 Years	7 (13)	4 (6)	2 (9)	13 (9)	0.21
During WCD Use	1 (2)	0 (0)	1 (4)	2 (1)	0.44
Overall	8 (14)	4 (6)	3 * (13)	15 (10)	0.13

* One Patient died after ICD—Explant; two because of myocarditis; ^1^ *p*-value for comparison of ICM versus NICM; ICM = ischemic cardiomyopathy; NICM = non-ischemic cardiomyopathy.

## Supplementary Materials

The following are available online at https://www.mdpi.com/2077-0383/9/3/893/s1, Table S1: Characteristics of patients with appropriate WCD shocks; Table S2: Characteristics of patients with appropriate ICD shocks; Table S3: Characteristics and variables of patients and frequencies for death and Table S4: Characteristics and variables of patients and frequencies for shocks.
