# Supplementary material for: The Wearable Cardioverter-Defibrillator: Experience in 153 Patients and a Long-Term Follow-Up
Source: J Clin Med. 2020 Mar 24;9(3):893. doi: 10.3390/jcm9030893 (PMC7141506; doi:10.3390/jcm9030893)
Supplement: Supplementary file 1 [file jcm-09-00893-s001.pdf]

Table S1. Characteristics of patients with appropriate WCD shocks.

Sex	Age	WCD Indication	LVEF at baseline	Days till WCD shock	Type of arrhythmia	WCD Shock successful	ICD implant.	Death during 3 yr follow up	Arrythmia during Follow up/ type of arrhythmia	ICD shocks
male	66	ICM	35	62	VT	yes	yes	no	VT	yes
female	31	Myocarditis	53	37	VT	yes	yes	no	no	
male	48	ICD-Explant. (ICM)	30	56	VT	yes	yes	no	no	
male	53	ICM	25	2	VF	yes	yes	no	no	
male	64	ICM	35	64	VT	yes	yes	no	VT	yes
male	75	ICM	20	151	VT	yes	yes	no	no	

WCD= wearable cardioverter-defibrillator, ICD= implanted cardioverter-defibrillator, LVEF = left ventricular function (%), VT= ventricular tachycardia;

VF= ventricular fibrillation, ICM = ischemic cardiomyopathy, NICM = non-ischemic cardiomyopathy

Table S2. Characteristics of patients with appropriate ICD shocks.

Sex	Age	Indication WCD	LVEF at baseline	improved LVEF after 6-12 months (>35%)	Shock while WCD	Type of arrhythmia	ICD shocks	Death during follow up
M	66	ICM	35	yes	yes	VT	y	n
M	63	ICM	30	no	no	VT	y	n
M	79	ICM	25	no	no	VT	y	n
M	54	NICM/Myocarditis	15	yes	no	VT	n	n
M	65	NICM	25	yes	no	VT	y	n
M	64	ICM	35	unknown	yes	VT	y	n
M	65	ICM	35	no	no	VT	y	n
M	74	ICD-Explant.	30	no	no	VT	y	n

WCD= wearable cardioverter-defibrillator; LVEF = left ventricular function (%); ICD= implanted cardioverter-defibrillator;
ICM = ischemic cardiomyopathy, NICM = non-ischemic cardiomyopathy; VT= ventricular tachycardia.

Table S3. Characteristics and variables of patients and frequencies for death.

Variables	death, n = 15	no death, n = 134
Age in years, mean ± SD	71 ± 12	59 ± 13
Male, n (%)	11 (73)	105 (78)
WCD wear days, mean ± SD	40 ± 26	68 ± 43
Former significant VT/VF* event prior to WCD* use	6 (40)	19 (14)
Arrhythmic episodes during WCD use, n (%)	0 (0)	6 (4)
LGE*, n (%)	2 (13)	57 (43)
LVEF* at baseline, mean ± SD	29 ± 14	29 ± 10
Elevated BNP at baseline, mean ± SD	7461 ± 6074	7778 ± 28160
QRS at baseline, mean ± SD	138 ± 42	107 ± 28
QTc at baseline, mean ± SD	480 ± 48	458 ± 56
PQ-time at baseline, mean ± SD	168 ± 16	165 ± 27
History of CAD*, n (%)	10 (67)	51 (38)
Dialysis as comorbidity, n (%)	6 (40)	14 (10)
Risk factor Diabetes, n (%)	7 (47)	24 (18)
Risk factor Hypertension, n (%)	12 (80)	72 (54)
Risk factor Active smoker, n (%)	4 (27)	61 (46)
Risk factor Lipidemia, n (%)	11 (73)	55 (41)
Risk factor Overweight, n (%)	9 (60)	95 (71)
Family history of CVD*, n (%)	1 (7)	40 (30)
ACE* inhibitors, n (%)	14 (93)	117 (87)
Aldosterone antagonists, n (%)t	7 (47)	78 (58)
Betablockers, n (%)	15 (100)	128 (96)

*VT, ventricular tachycardia; VF, ventricular fibrillation; WCD, wearable cardioverter defibrillator; LGE, late gadolinium enhancement; LVEF, left ventricular ejection fraction; CAD, cardiac artery disease; CVD, cardiovascular disease; ACE, angiotensin converting enzyme

Table S4. Characteristics and variables of patients and frequencies for shocks.

	**Patients with shocks, n = 8**	**Patients without shocks, n = 54**
Age in years, mean ± SD	67 ± 7	60 ± 14
Male, n (%)	8 (100)	41 (76)
WCD wear days, mean ± SD	63 ± 32	65 ± 41
Former significant VT/VF* event prior to WCD* use	1 (13)	12 (22)
Arrhythmic episodes during WCD use, n (%)	2 (25)	4 (7)
LGE*, n (%)	5 (63)	16 (30)
LVEF* at baseline, mean ± SD	29 ± 7	29 ± 11
Elevated BNP at baseline, mean ± SD	4001 ± 2628	8264 ± 28892
QRS at baseline, mean ± SD	117 ± 35	108 ± 30
QTc at baseline, mean ± SD	405 ± 141	462 ± 44
PQ-time at baseline, mean ± SD	176 ± 30	164 ± 26
History of CAD*, n (%)	5 (63)	26 (48)
Dialysis as comorbidity, n (%)	2 (25)	8 (15)
Risk factor Diabetes, n (%)	1 (13)	12 (22)
Risk factor Hypertension, n (%)	6 (75)	36 (67)
Risk factor Active smoker, n (%)	6 (75)	23 (43)
Risk factor Lipidemia, n (%)	6 (75)	31 (57)
Risk factor Overweight, n (%)	5 (63)	36 (67)
Family history of CVD*, n (%)	1 (13)	13 (24)
ACE* inhibitors, n (%)	8 (100)	45 (83)
Aldosterone antagonists, n (%)t	3 (38)	33 (61)
Betablockers, n (%)	8 (100)	53 (98)
